# Searching for Life on Mars Before It Is Too Late

**DOI:** 10.1089/ast.2017.1703

**Published:** 2017-10-01

**Authors:** Alberto G. Fairén, Victor Parro, Dirk Schulze-Makuch, Lyle Whyte

**Affiliations:** ^1^ Centro de Astrobiología (CSIC-INTA), Madrid, Spain.; ^2^ Department of Astronomy, Cornell University, Ithaca, New York.; ^3^ Center of Astronomy and Astrophysics, Technical University Berlin, Berlin, Germany.; ^4^ SETI Institute, Mountain View, California.; ^5^ Department of Natural Resource Sciences, McGill University, Québec, Canada.

## Abstract

Decades of robotic exploration have confirmed that in the distant past, Mars was warmer and wetter and its surface was habitable. However, none of the spacecraft missions to Mars have included among their scientific objectives the exploration of Special Regions, those places on the planet that could be inhabited by extant martian life or where terrestrial microorganisms might replicate. A major reason for this is because of Planetary Protection constraints, which are implemented to protect Mars from terrestrial biological contamination. At the same time, plans are being drafted to send humans to Mars during the 2030 decade, both from international space agencies and the private sector. We argue here that these two parallel strategies for the exploration of Mars (*i.e.*, delaying any efforts for the biological reconnaissance of Mars during the next two or three decades and then directly sending human missions to the planet) demand reconsideration because once an astronaut sets foot on Mars, Planetary Protection policies as we conceive them today will no longer be valid as human arrival will inevitably increase the introduction of terrestrial and organic contaminants and that could jeopardize the identification of indigenous martian life. In this study, we advocate for reassessment over the relationships between robotic searches, paying increased attention to proactive astrobiological investigation and sampling of areas more likely to host indigenous life, and fundamentally doing this in advance of manned missions. Key Words: Contamination—Earth Mars—Planetary Protection—Search for life (biosignatures). Astrobiology 17, 962–970.

## 1. Introduction

The main reason that international space agencies adduce to continue investing billions of dollars in Mars exploration is its potential for life and astrobiology. We concur that the biological exploration of Mars would find little match among the major scientific objectives for upcoming decades. Unfortunately, with the exception of the two Viking landers in 1976, all other lander and rover missions to Mars have been in fact primarily geology focused, although they are often put forward as astrobiology missions. In this context, they have been quite successful in confirming that in the distant past, Mars was warmer and wetter and the surface was habitable (Squyres *et al.*, 2008; Arvidson *et al.*, 2014; Grotzinger *et al.*, 2014), but none of them carried true life detection instrumentation as Viking did, and when new missions are designed to incorporate a true life-searching payload, as for example, ESA's ExoMars Rover, they do not include among their objectives the exploration of the Special Regions, defined as the places on Mars where terrestrial microorganisms might replicate or could be inhabitable by extant martian life.

This paradox arises from the implementation of Planetary Protection policies (Box 1). The United Nations Outer Space Treaty stipulates protection of targeted celestial bodies to prevent forward contamination to avoid their harmful contamination (United Nations Office for Disarmament Affairs, 2015), and the COSPAR Planetary Protection policy statement proclaims that the conduct of scientific investigations of possible extraterrestrial life forms, precursors, and remnants must not be jeopardized (Rummel *et al.*, 2014; NASA Planetary Protection Office, 2015; COSPAR, 2016). In general, these Planetary Protection constraints have been largely interpreted to be in place to protect Mars from terrestrial biological contamination, safeguarding a possible martian biosphere, and to protect scientific investigations—for the mission at hand *and* for future missions—thus assuring that future generations could eventually study martian microorganisms without concerns of a potential detection of microbes carried forward on our spacecraft today, resulting in false-positive results.

Box 1. The Evolving Planetary Protection Policies *Planetary Protection* requirements are not static. There has been a continuous historical development of views on Planetary Protection and its categories as applied to Mars, which have particularly changed considerably since the Viking missions. There have been also multiple international studies and deliberation on the matter of Planetary Protection categories and approaches since Viking, particularly between Viking and Pathfinder missions. Additional changes are required at this moment, particularly because of the needed interaction with the Mars manned mission community, as highlighted in this article.

*Special Regions* were not discovered and walled off overnight. Special Regions were first discussed at the 2002 COSPAR Workshop, then later in more detail by MEPAG in 2006, COSPAR in 2007, MEPAG in 2104, and in a comprehensive review by NRC-ESA in 2015. Collectively, these studies integrated the knowledge and opinions of a large community of astrobiologists from multiple disciplines as well as international policy contributors, who reviewed voluminous data about Planetary Protection categories, extremophiles, and Mars.

*Bioburden reduction requirements* have also changed over time, from pre- and post-Viking. Updates to Planetary Protection requirements have resulted from additional advances in understanding about microbes and microbiomes in general, cleanroom technologies, new nonculture methods of detecting microbes, increasing knowledge of extremophiles on Earth, and better understanding of martian environments over time. Like any regulatory standards and implementations, the Planetary Protection levels and enactment protocols are set based on consensus of scientific input and balances with other needs.

Although no areas on Mars are theoretically off-limits to exploration as long as the missions meet the applicable contamination constraints, the reality is that current Planetary Protection policies are based on such stringent microbial reduction efforts for a life-searching mission (Rummel *et al.*, 2014) that, in practice, they have become a cost-prohibitive benchmark (Fairén and Schulze-Makuch, 2013) that is barring the implementation of strategies to search for life in the Special Regions.

It could be argued that a future proper life detection mission would not necessarily face problems in the exploration of Special Regions because it would have already set cleanliness standards that exceed the level of today's requirements. However, in our opinion, that would be wishful thinking. The reality is that the restrictions go to the extreme such that if the current NASA's Curiosity or the upcoming NASA's Mars2020 and ESA's ExoMars rovers came close to a Special Region, they would not be allowed to use their considerable (costly and difficult-to-put-there) instrumentation to sample and analyze for potential biosignatures because they are not cleaned to appropriate levels.

The best example of this is Curiosity (Benardini *et al.*, 2014), which was recently forbidden (Witze, 2016) to attempt to sample and analyze readily accessible recurring slope lineae (RSLs, narrow streaks formed on the surface arguably as a result of contemporary water activity, see Ojha *et al.*, 2015; Edwards and Piqueux, 2016), where the instruments onboard would have been able at least to test whether they contained briny liquid water; as a result, we will have to put together another multibillion dollar mission (with a 40% chance of landing successfully, see the latest Schiaparelli attempt) to essentially do what Curiosity could do presently in Gale Crater. NASA's Mars2020, which will introduce a drill to collect core samples of rocks and soils to search for signs of past microbial life (NASA Mars2020 mission overview), has likewise been directly required to avoid landing in Special Regions because it also will not be cleaned to appropriate levels.

The case of ExoMars is particularly dramatic as the first priority of the rover is to search for signs of past and present life on Mars (ESA's Scientific objectives of the ExoMars Rover, 2016); however, it has been explicitly banned to go to Special Regions because it will not comply with the minimum cleanliness requirements. As a consequence, the billion-dollar life-seeking mission ExoMars will be allowed to search for life everywhere on Mars, except in the very places where we suspect that life may exist. This incongruous situation has been stagnant for a long time and has delayed, *sine die*, a real quest for life on Mars.

However, now there is a game changer: after years of timid insinuations, NASA is, for the first time, seriously planning to send humans to Mars after 2030 (First Landing Site/Exploration Zone Workshop for Human Missions to the Surface of Mars, 2015; Obama, 2016; National Aeronautics and Space Administration Transition Authorization Act, 2017), including chartering a previous planning study to support martian water *in situ* resource utilization for eventual human missions (MEPAG, 2016). In addition, given the rapid advances in space flight technologies by other national space programs as well as within the private sector, it is not out of the realm of possibilities that other stakeholders may precede NASA in completing human missions to Mars (Musk, 2017), and the moment that an astronaut sets foot on Mars, Planetary Protection policies as we conceive them today will no longer be valid as microbial contamination from the human visitors will be unavoidable (McKay, 2009; Siefert, 2012); humans will increase not only the number (a human being is a collection of roughly 70 trillions of cells and bacteria; however, microbial invasion is not simply a matter of numbers) but also most importantly the diversity of microorganisms flying to Mars.

In addition, those microorganisms associated with the hardware will never be removed, which, along with the hardware, will be traveling anyway. All this microbial diversity would potentially leak out of a spacecraft or habitat module or waste deposit, and some of the organisms could end up in Special Regions because of transport by wind. In addition, *in situ* resource utilization, with the aim of extracting and processing martian resources to obtain life support consumables and propellants, will enormously raise the chances of forward contamination, especially during soil processing to extract liquid water.

The list of knowledge gaps that we need to address to begin to understand the actual contamination risks posed by a manned mission to Mars is challenging (NASA Workshop on Planetary Protection Knowledge Gaps for Human Extraterrestrial Missions, 2015; NASA Policy on Planetary Protection requirements for human extraterrestrial missions, 2016; National Academies, 2016). Therefore, the current strategy for the exploration of Mars, in practice, delays any efforts for the biological reconnaissance of Mars during the next two or three decades due to Planetary Protection concerns, but then, to subsequently send human missions directly to the planet would seem an unfortunate approach that without doubt would tremendously complicate our quest for indigenous life on Mars in the future.

We argue that the space science community should explore Mars thoroughly from a biological point of view over the next 10 to 20 years. To do so, we recommend that Planetary Protection restrictions are substantially relaxed to facilitate our robots and dedicated instruments to access and investigate the Special Regions. We argue that the benefits of this approach would offer significantly more with regard to planetary science research than any possible detriments from it by (1) facilitating discussion as to whether forward biological contamination of the surface of Mars by robots is so unlikely that the current stringent Planetary Protection policies deserve a serious rethinking and (2) acknowledging that we have the capability to distinguish a martian microorganism from that which is terrestrial when searching for life on Mars. We recommend this urgent and sharp turn of direction in Mars exploration because the alternative of remaining passive while waiting to see astronaut footprints on the red martian soil will very soon close off options for the future biological reconnaissance of Mars, putting us at a point of no return.

## 2. The Unlikely Contamination of Mars

We know that microbial life has already been transferred from Earth to Mars on more than one occasion, either naturally through impact events or through partially or nonsterilized spacecraft that have landed or crashed on Mars during the last five decades. Therefore, if Earth microorganisms can, in fact, survive and create active microbial ecosystems on present-day Mars, we can presume that they are already there; on the other hand, if Earth life cannot survive and most importantly reproduce on Mars today, our concerns about forward contamination of Mars with terrestrial organisms are unwarranted (Fairén and Schulze-Makuch, 2013).

The survival of Earth microorganisms on the surface and shallow subsurface of Mars is very unlikely (Pavlov *et al.*, 2002; Nicholson *et al.*, 2009; Khodadad *et al.*, 2017). We have been sending dirty spacecrafts to Mars since the Viking missions in 1976, yet compliant with the bioburden requirements based on scientifically accepted standards and protocols. Even the cleanest of missions carried hundreds of thousands of microbial stowaways, simply because we do not know how to completely sterilize our probes (*e.g.*, Viking-level dry heat microbial reduction is often incorrectly termed as sterilization, when it is not, see Nicholson *et al.*, 2009).

In addition, unfortunately, the organisms that survived our cleaning procedures are actually the most hardy ones because they are more resistant to some of the same stresses that they are later exposed to on the surface of Mars (*e.g.*, UV irradiation, plasma, oxidizing chemicals such as vapor hydrogen peroxide, and heat microbial reduction); therefore, we suggest that future research may show that current cleaning protocols are essentially conducting an artificial selection experiment, with the result that we carry on our spacecrafts only those microorganisms that may have a chance to survive on Mars—the others would be of no concern anyway—putting into question the whole cleaning procedure.

However, the surface of Mars has been, and still is, methodically sterilized with a broad radiation spectrum, extreme cold and dryness, and an inhospitable surface soil chemistry in the form of highly reactive oxidizing agents that essentially destroy most organic molecules (Nicholson *et al.*, 2009; Freissinet *et al.*, 2015; Khodadad *et al.*, 2017). It is true that multitudes of extremophile microbiology studies on Earth have demonstrated the extraordinary capacity of terrestrial microorganisms to create active microbial ecosystems in a variety of extreme terrestrial environments on Earth. In addition, numerous studies have shown the survivability of Earth organisms in Mars simulation chamber experiments up to months in artificial martian surface regolith (*e.g.*, de Vera *et al.*, 2013; Smith *et al.*, 2017). However, the only report to date that hints of potential microbial growth under martian surface conditions demonstrated reproduction of Siberian *Carnobacterium* isolates under cold, low-pressure, and anoxic conditions (Nicholson *et al.*, 2013), although this assay was conducted in a rich growth medium that would never occur in martian surface soils.

Therefore, there is still no convincing report that clearly demonstrates how terrestrial microorganisms would survive and, crucially, be able to reproduce and form active microbial communities on the surface of Mars. If we were to stage a mission to Mars to investigate one of our non- or partially sterilized previous landers/rovers and reexamine it for life, we would anticipate that the outside of the spacecraft would be sterile due to several years of exposure to the surface environment of Mars, much more so compared with when the spacecraft left Earth (eventually, microbes inside the metal spacecraft will be killed by cosmic rays too, but that will take much longer than a few years).

Some recent investigations support the unlikely survival of earthlings on Mars. First, a motorized expedition was recently conducted in the Arctic to gain experience about future road trips on Mars (Schuerger and Lee, 2015). Along the way, samples of grit and snow were collected from inside and outside the vehicles to investigate whether (and the extent to which) microbes transported by the vehicles and crew might have found their way onto the surrounding pristine snow surface outside the vehicle. The results strongly indicate that in an environment immensely less harsh for terrestrial microbes than Mars, forward contamination was extremely limited to nonexistent.

Second, we have just learned that in an upper Antarctic Dry Valley near surface soils, where the ice in permafrost originates from vapor deposition rather than liquid water (similar to martian near-surface permafrost environments), microbial activity and survival are strictly limited because of the combination of severe cold, aridity, and oligotrophy (Goordial *et al.*, 2016). If soils in the upper Dry Valleys are potentially uninhabitable, even when they are continuously seeded with allochthonous organisms through aeolian deposition, then it is difficult to envision how Mars surface environments could ever support active microbial ecosystems, resulting from a handful of terrestrial microorganisms hitchhiking their way to Mars, because martian surface permafrost environments are much colder, drier, and older; receive much more radiation; and receive much less input (if any) of organic C, N, P, or allochthonous organisms than the upper Dry Valleys.

All the previous arguments have been tested, and we have strong evidence to claim that biological contamination has not occurred at global scales on the surface of Mars; the Curiosity rover is well equipped to identify organic compounds and still is having a very difficult time finding some (Freissinet *et al.*, 2015). Hence, if any terrestrial hitchhikers survived the journey to Mars and are still alive on the surface of the planet, they would be hiding within or around our shipwrecks and not planning field trips. Factoring in the potential natural transfer of microbes through meteorites, this argument can be extended back for millions or even billions of years. Therefore, even if terrestrial microorganisms were transported to Mars in one or several occasions during millions or billions of years, it is highly unlikely that there is a global biosphere derived from Earth organisms on the surface of Mars today, so the contamination (if any) has been very limited in space and time (McKay, 2009).

## 3. Recognizing the Martians

A different question would be the possibility of false positives, that is, any potential microbial hitchhikers on our spacecrafts could be mistaken for martian life. Should this really be a concern? We think it should not because molecular biology has advanced considerably in the last decades, and new methods in laboratory analysis make false-positive results far less likely. As such, if we find microorganisms on Mars by detecting their molecular markers (proteins, pigments, polysaccharides, and nucleic acids), we should be able to extract and sequence their genetic material to investigate their origin (*in situ* and/or analyzing returned samples). Indeed, we only consider here the case that the martians are biochemically similar enough to Earth life that such mistakes could be a concern; if the martians have a different system to store and transmit their genetic information, then by definition, there will be no chance of mistakes in the identification (McKay, 2008). The arguments hereafter are more speculative as our intention is to open new avenues for this debate.

The DNA/RNA sequence is as specific as a personal passport and could reveal the roots and origin of the organisms. As sequencing complete genomes would prove problematic, and the existing databases are not yet large enough, 16S/18S ribosomal RNA sequencing will possibly be the better choice. If it is just contamination, multiple organisms should be found all very closely related to others (*i.e.*, the same level of similarity typically identified when new Earth isolates are sequenced) in the database. This way, we may be able to map potential microbes found on Mars onto the universal tree of life and identify them. We do this routinely in our laboratories, and we are able to separately map very similar strains in different positions on the tree of life. As it is expected that the RNA/DNA sequence of any potential martian microorganisms will be significantly different from any organism on Earth, indigenous martian microbes might form a new kingdom or subkingdom at the very base of the Bacteria or Archaea branches of the universal tree of life. Furthermore, if panspermia occurred in the distant past, then 16S rRNA sequences from Mars organisms would consistently map to various subgroups on the tree of Earth life, but never to specific modern organisms. Depending on the level that this occurs (genus, order), the data would supply information on when the transport had occurred.

**FIG. 1.**
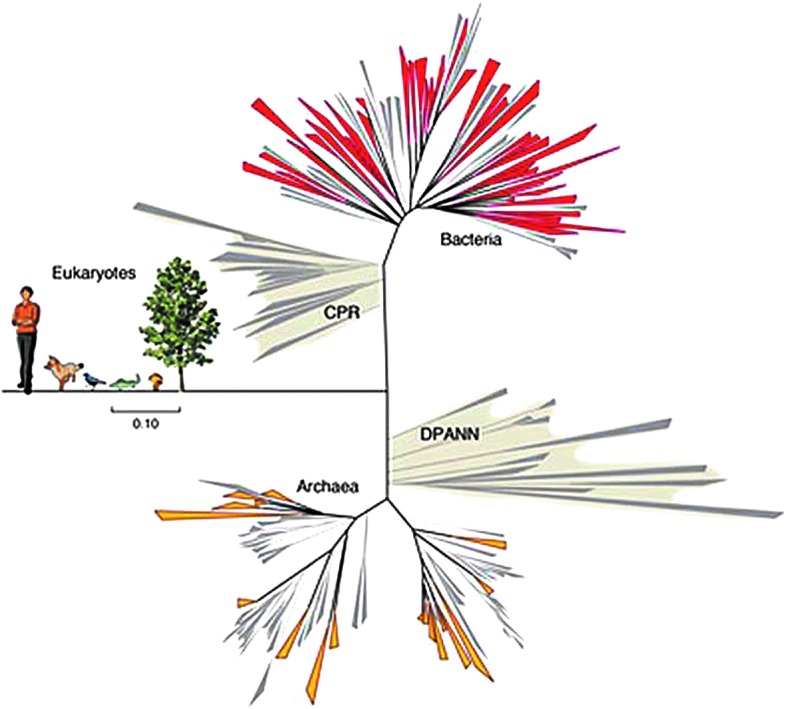
The new groups of Bacteria and Archaea discovered in 2015 (CPR and DPANN, respectively) greatly expand the known and characterized phyla in a more and more complex tree of life, in which entire new branches are still being identified. These new advances show that we will be knowledgeable enough as to know where to distinctly map in the tree of life potential microorganisms found on Mars. (Image courtesy J. Banfield). CPR, Candidate Phyla Radiation; DPANN, Diapherotrites, Parvarchaeota, Aenigmarchaeota, Nanohaloarchaeota, and Nanoarchaeota.

As an analog to Earth investigations, hundreds of new types of microorganisms were recently found in understudied terrestrial environments and they ended up comprising more than 15% of all known groups of Bacteria and nine new groups of Archaea, representing tens of new phyla about which very little was previously known (Brown *et al.*, 2015; Castelle *et al.*, 2015) (Fig. 1). These new phyla were found to occupy entire new branches on the tree of life (Hug *et al.*, 2016).

Another example that demonstrates our ability to identify and trace novel organisms occurred during the 2013 outbreak of the bacterium *Xylella fastidiosa* in olive trees in Italy; microbiologists traced the particular strain of *Xylella* involved in the Italian outbreak as endemic in Costa Rica, the single place in the World where the strain was previously identified, and determined that the disease arrived in Italy with ornamental plants imported from Costa Rica (Abbott, 2015). Since we know how to attain such a high level of specificity in identifying the origin of terrestrial organisms, we may be able to identify and trace Earth life forms transported to Mars because they would be genetically much more separated from potential martian microorganisms.

The differences between martian and terrestrial organisms may eventually go beyond biochemistry, including their very cellular structure. If we find Earth-like life on Mars, having originated on Mars or having been transferred from Earth billions of years ago, this martian biosphere would have been exposed to the geological and environmental evolution of Mars, which was extremely different from that of Earth at least during the last 3 billion years. As a consequence, the evolutionary traits of these martians would be expected to be very different from indigenous Earth organisms.

For example, possibly some of the great events in Earth's life evolution, such as the endosymbiotic inception of the mitochondria and chloroplast from bacterial precursors, may never have occurred on Mars and hence only prokaryotes might have proliferated and evolved. In addition, bearing in mind that eukaryotes did not appear on Earth until ∼2 Gyr ago (Rasmussen *et al.*, 2008) and multicellular life until ∼1.5 Gyr ago (Zhu *et al.*, 2016), and considering that Mars has been a very cold and dry planet since at least 3 Gyr ago (*e.g.*, Fairén, 2010), the replication rate and hence the evolutionary pace of any possible martian biota may have been very slow, and therefore eukaryotic organisms might not exist on Mars.

**FIG. 2.**
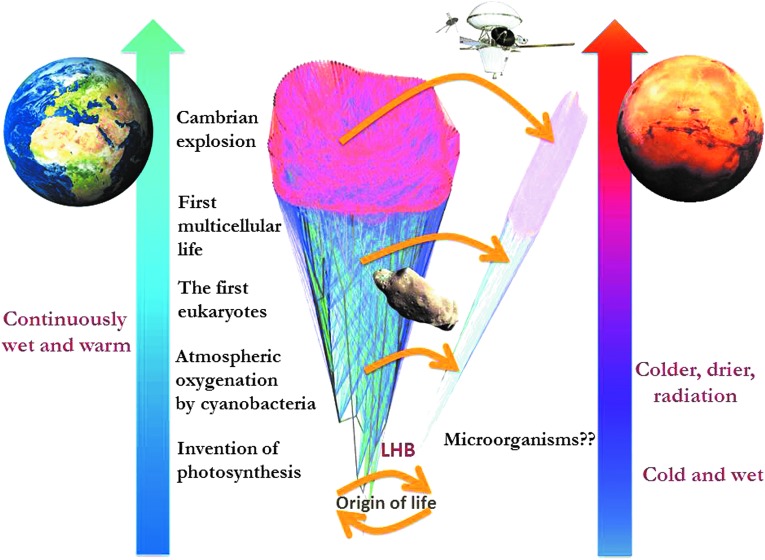
Major evolutionary events of life on Earth represented together with the possible trajectory of a hypothetical martian biosphere. The origin of life could have occurred on Earth, on Mars, or on both planets, and then transferred from one to the other. On Earth, life gained a foothold early on and started to diversify (represented by the fat cone), driven by genetic interchange through promiscuous horizontal gene transfer (represented by the lines in the cone), and to transform the planet. The possible biological history of Mars is totally unknown (represented by the hypothetical thinner and blurred cone), maybe including scarce horizontal gene transfer events, resulting in smaller phylogenetic groups. The yellow arrows represent possible natural transfer of microbes from Earth to Mars through meteorites, a common event throughout the entire history of the Solar System. Today, the concern seems to be the possible presence of hitchhikers onboard our spacecrafts. LHB = Late Heavy Bombardment.

Terrestrial eukaryotes would have had infrequent transport to Mars as they appeared in a time with already reduced rock interchange between the planets; however, more importantly, those that were transported to Mars would have found there an extremely hostile environment totally different than their home planet. As a result, it is possible that only primitive life forms succeeded on Mars, such as lithoautotrophs, heterotrophic bacteria, and cyanobacteria, with neither relation to more modern terrestrial life forms nor horizontal genetic exchange with Earth's biosphere. A divergent evolution of biospheres on Earth and Mars may have the result that simple morphological and structural analyses would reveal noticeable differences between the martian and terrestrial microbiota (Fig. 2).

Using the same reasoning the other way around, if we identify life on Mars and it turns out to be genetically and biochemically very similar or identical to Earth life, then we can reasonably presume that we have caught our hitchhikers from Earth under the microscope. It is biologically and evolutionarily difficult to suggest that indigenous (or very early transported from Earth) martian life would be genetically and structurally so similar to modern Earth life that we would risk making wrong identifications. In addition, RNA/DNA analyses will be the perfect tool with which to identify the origin of potential organisms found on Mars. Furthermore, if putative martian organisms are found with no relative in the universal tree of life, then it would be regarded as likely martian, when it might just be related to members of a not yet discovered branch in the tree of Earth Life. However, since we would typically be looking at populations, finding a single organism in a population with no relatives could be explained as just a *not yet* described Earth organism.

It is true that RNA/DNA sequencing of martian samples would be challenging. Assuming the hypothetical case that some terrestrial cells transported by spacecrafts were able to survive and reproduce on Mars, their growth rate and generation time (the time required to double the number of cells in a microbial population) at freezing or nearly freezing temperatures (ambient conditions on Mars) would be extremely slow. For example, *Planococcus halocryophilus*, a permafrost bacterium with the coldest growth temperatures yet reported, was capable of doubling in ∼40 days at −15°C (Mykytczuk *et al.*, 2013); however, this assay was performed in a rich growth medium under optimal laboratory conditions, which would likely be much greater than would be found under ambient permafrost conditions.

A more realistic example would be the generation time of 2.5 years for bacteria exposed to temporal freeze–thaw cycles in the permanent ice covers of Antarctic lakes (Fritsen and Priscu, 1998). Assuming such an optimal environmental situation for Mars, a contamination of 100 metabolically active cells would require 50 years to produce a cell density of about 5000 cells/g in a square kilometer. This is in the threshold of many life detection systems, but enough to recover RNA/DNA in a sample return mission or to amplify the nucleic acids with polymerase chain reaction (PCR) techniques both *in situ* and back on Earth.

Furthermore, we have an excellent control with which to monitor the potential contamination of Mars: sequencing the microbes found in the clean spacecraft assembly rooms (Moissl-Eichinger *et al.* 2012; Checinska *et al.*, 2015; van Heereveld *et al.*, 2016). Any sequence identical or highly similar to those found on a martian sample would indicate very likely contamination and should be discarded as being indigenous to Mars.

All the facts described above strongly suggest that if we ever find microorganisms on Mars, we will be knowledgeable enough to distinguish martian (exobiota) from terrestrial (contamination) life. That of course applies only for a short time span in the future, while the terrestrial biological contamination of Mars (if any) remains contained (close to our spacecraft) and known (present in our clean rooms) and therefore manageable. Human missions will change the name of that game forever.

## 4. A New Road Map for Mars Exploration in the 21st Century

Following the heritage of the Viking landers (the only true life detection mission to Mars so far), we urge resumption of a dedicated astrobiological policy such that we invest the adequate amount of resources and put them in the right place in advance of human missions. It is imperative that the Mars astrobiological and manned mission communities work closer together on the path forward, finding collaborative solutions for shared problems and including in the conversation scientists, managers, and policy makers. We enthusiastically support any efforts directed to the human exploration of Mars and so the alternative of halting the advance of manned missions would not be a solution for us. Therefore, if we, the Mars community, are truly committed to determine whether life ever existed or still exists on Mars, we propose here a twofold change of strategy.

First, we advocate allowing immediate access to the Special Regions for vehicles with the cleanliness level of Curiosity, Mars2020, or ExoMars. For this, it would be necessary to reevaluate the current Planetary Protection restrictions and make sure they are properly adapted for the new space age we are entering, particularly distinguishing clearly between spacecraft cleanliness for biological reconnaissance and spacecraft cleanliness for planetary protection. This will reduce the likelihood that spacecraft cleanliness issues create conflicts between planetary protection efforts and science objectives, abiding to the principle that Planetary Protection policies should enable the exploration of Mars and not prohibit it (COSPAR Panel on Planetary Protection, 2017).

These proposed changes would require that COSPAR update the rules governing the robotic exploration of Mars at its next meeting in 2018, and the Outer Space Treaty should be amended as well. As immediate prospective go-to places, we particularly recommend analyzing potential transient liquid aqueous solutions and water ice, such as those recently identified in (1) the RSL (Ojha *et al.*, 2015); (2) the nighttime ephemeral brines formed in the shallow subsurface by absorbing atmospheric water vapor through deliquescence (Martín-Torres *et al.*, 2015); and (3) the shallow subsurface water ice at mid-high latitudes, where SHARAD radar data suggest that large layers, decameters thick, would enclose volumes of 10^4^ km^3^ of water (Bramson *et al.*, 2015; Stuurman *et al.*, 2016).

Second, we urge that our existing laboratory robotic technology is made flight ready in the search for biochemical evidence of life (McKay *et al.*, 2013; Schulze-Makuch *et al.*, 2013), and in particular, we advocate the development of robotic nucleic acid sequencing instrumentations for future *in situ* detection and/or sample return (Carr *et al.*, 2013). We will need parallel analyses for complex and polymeric sugars, lipids, peptides, and nucleic acids, as well as their building blocks such as sugars, nucleobases, and amino acids, so we will no longer be concerned about possible false-positive life detection. Robotic microscopes with very high resolution to analyze samples could also help to identify different cellular architectures.

The immediate (*i.e.*, in less than 10 years) implementation of this new strategy outlined here is vital: before any human mission lands on Mars and exposes the planet to an unprecedented and very likely irreversible level of terrestrial bioburden, we should determine with well-designed life detection experiments whether any indigenous life exists on Mars, at least close to the anticipated human landing site and especially where we suspect that life might thrive (at the Special Regions nearby). We urge the prompt adoption of a proactive and comprehensive astrobiological strategy to find extant martian life before the onset of human missions, as opposed to continuing our sending more and more robotic geologists to Mars' sites where we do not expect the presence of life and, all the while, delay the biological exploration of the planet.

What we highlight here is a problem of timing: if we had still 50 or 70 years with no forecasted human presence on Mars ahead of us, we could sympathize with more conservative approaches for searching for extant martian life, but manned missions are already planned and budgeted for less than 20 years from today. It is very likely that our children or grandchildren (the Mars generation; Obama, 2016) will see astronaut footprints on the red sands of Mars, and at that moment, it will be much too late to straightforwardly identify the nature of true indigenous martians. This would be a lamentable loss for science because the main goal of Mars exploration should be to try and find life on Mars, understand the biochemical nature of martian life, and compare it with terrestrial life. Any scientifically rigorous search for life and understanding of its nature on Mars must address the question of whether it is a separate genesis or shares a common ancestor with life on Earth. Finding signs of life on Mars should not be the end or the mission accomplished coda for decades of planetary investigation; on the contrary, it will be just the beginning of a new age for science, culture, philosophy, and exploration.

## Abbreviations Used

CPR: Candidate Phyla Radiation
DPANN: Diapherotrites, Parvarchaeota, Aenigmarchaeota, Nanohaloarchaeota, and Nanoarchaeota.
PCR: Polymerase Chain Reaction
RSL: Recurring Slope Lineae
